# Analysis of Exercise Intensity Preferences, Tolerance, Competence, and Their Implications for Behavioral Intentions in Fitness Settings

**DOI:** 10.3390/jfmk8030139

**Published:** 2023-09-20

**Authors:** Filipe Rodrigues, Miguel Jacinto, Raúl Antunes, Nuno Amaro, Rui Matos, Diogo Monteiro

**Affiliations:** 1 ESECS—Polytechnic of Leiria, 2411-901 Leiria, Portugal; filipe.rodrigues@ipleiria.pt (F.R.); miguel.s.jacinto@ipleiria.pt (M.J.); raul.antunes@ipleiria.pt (R.A.); nuno.amaro@ipleiria.pt (N.A.); rui.matos@ipleiria.pt (R.M.); 2 Life Quality Research Centre (CIEQV), 2400-901 Leiria, Portugal; 3 Center for Innovative Care and Health Technology (ciTechCare), 2415-396 Leiria, Portugal; 4 Research Center in Sport, Health, and Human Development (CIDESD), 5000-558 Vila Real, Portugal

**Keywords:** intensity traits, Self-Determination Theory, exercise, clients

## Abstract

This study aims to comprehensively investigate the interrelationships among exercise intensity preference, tolerance, competence satisfaction, competence frustration, and exercise intentions within the fitness domain. The research involved 114 participants aged 18 to 59 years (M = 33.23; SD = 10.542), with an average height of 166.02 cm (SD = 15.856) and weight of 68.02 kg (SD = 13.658). The path analysis revealed positive correlations among constructs, except for the link between exercise intensity tolerance and competence satisfaction. Notably, exercise intensity preference positively related to competence satisfaction (β = 0.20, CI90% = 0.12, 0.40), while both preference and tolerance were negatively linked to competence frustration (preference: β = −0.27, CI90% = −0.44, 0.03; tolerance: β = −0.17, CI90% = −0.03, 0.24). Competence satisfaction (β = 0.37, CI90% = 0.13, 0.34) and competence frustration (β = −0.29, CI90% = −0.48, −0.09) significantly influenced exercise intentions. An indirect effect existed between exercise intensity preference and intentions (β = 0.11, CI90% = 0.03, 0.21), contrasting with the lack of such effect for intensity tolerance (β = 0.05, CI90% = −0.01, 0.14). Intentions explained 16% of the variance. In conclusion, this study underscores the intricate connections between exercise intensity traits and intentions, shedding light on factors influencing individuals’ exercise persistence.

## 1. Introduction

In the realm of fitness, understanding the factors that underpin the continued engagement of existing gym-goers remains a compelling pursuit of research [1]. The endeavor to comprehend the intricate fabric of variables that exert influence over the continuation of exercise regimens among this demographic has captivated the attention of researchers and practitioners alike. Among these variables, the dimensions of exercise intensity, preference, and tolerance stand out as essential determinants of the exercise experience [2,3]. Notably, these aspects are particularly pertinent to individuals who have already embraced gym-based exercise routines. The interplay between an individual’s initial leanings towards exercise intensity and the concurrent cultivation of exercise experiences takes on a significant role in shaping not only engagement and satisfaction, but also the formulation of exercise intentions [2,4,5,6]. Concurrently, the pivotal role of perceived competence assumes prominence, as individuals’ self-appraisals of their capabilities significantly influence their exercise intentions [4,7,8].

Amidst individuals engaged in health and fitness-oriented exercise, substantial deviations from the recommended range of intensity, whether exceeding or falling short, can yield unfavorable outcomes [9,10]. Exceeding the suitable intensity might culminate in overexertion, injury, or discomfort, potentially deterring subsequent physical activity participation [11]. Conversely, opting for an intensity level beneath the prescribed range may impede the acquisition of discernible health and fitness advantages, giving rise to frustration and, once again, fostering the likelihood of discontinuation [9].

As emphasized by Ekkekakis et al. [5,12,13], the domain of exercise is characterized by notable divergence among individuals concerning their desired exercise intensity and their capacity to endure it. This intrinsic variation profoundly shapes the extent to which exercise can elicit an optimal affective response, a phenomenon that holds pivotal significance in the context of exercise adherence. The interplay between intensity and affective responses experienced during exercise sessions has garnered attention from scholars, with evidence suggesting a link to subsequent engagement in physical activity [14,15,16], thereby highlighting the pertinence of this relationship. Aligned with these foundational principles, scholars [5,12,13] have underscored the critical nature of exercise intensity preference and tolerance as variables shaping exercise experiences [4,5]. Exercise intensity preference encapsulates the distinct levels of exertion individuals aspire to during their workout routines. Concurrently, exercise intensity tolerance pertains to individuals’ ability to endure and sustain specific intensity levels throughout their exercise sessions.

Individuals’ exercise intensity preference and tolerance significantly influence their perceived competence and efficacy during exercise, creating a dynamic interplay. When exercise intensity aligns with their preference and tolerance levels, individuals are more likely to feel capable and proficient in their workouts, fostering a sense of competence and enhancing their self-efficacy. Conversely, a mismatch between preference, tolerance, and intensity might undermine their perceived abilities, potentially leading to increased feelings of competence frustration and reduced efficacy in the exercise context.

Within the framework of Self-Determination Theory, competence is regarded as one of the basic psychological needs that drives human behavior and well-being [17]. Competence refers to the intrinsic desire to feel effective, capable, and proficient in one’s actions and interactions with the environment, which extends to the exercise context [18]. Competence satisfaction in the fitness context corresponds to the sense of achievement and contentment experienced when individuals perceive themselves as skilled and capable in their exercise endeavors. On the other hand, competence frustration arises when individuals encounter situations that challenge their sense of mastery and efficacy. This might manifest when exercise intensities are misaligned with their preferences and tolerances, causing feelings of inadequacy or inability to meet the demands [18]. Conversely, competence frustration emerges when individuals encounter challenges or discrepancies that undermine their perceived mastery and efficacy in the exercise domain. The distinctiveness between competence satisfaction and competence frustration lies in their emotional and motivational outcomes. Competence satisfaction nurtures a positive affective state, reinforcing individuals’ sense of effectiveness and fostering a self-propelled drive to partake in exercise activities. In contrast, competence frustration engenders negative affect and undermines feelings of capability, potentially instigating extrinsic motivations or avoidance tendencies. When individuals experience competence satisfaction through aligned exercise preferences and tolerances, a positive loop of motivation is initiated. This sense of accomplishment and effectiveness engenders an internal drive, bolstering their intention to persistently engage in fitness activities [19,20]. Conversely, competence frustration, stemming from incongruence between preferences, tolerances, and exercise intensities, can undermine this motivation. Feelings of inadequacy might diminish the intention to continue or even initiate exercise, as the perceived difficulties erode the confidence required for sustained engagement [18,21].

In this study, we chose to focus specifically on the variables of exercise intensity preference, tolerance, and competence, and their impact on exercise intentions. While autonomy and relatedness are indeed important components within Self-Determination Theory, their theoretical relationship with exercise intensity preference and tolerance differs from that of competence. We opted not to measure autonomy satisfaction and frustration and relatedness satisfaction and frustration due to the nuanced theoretical distinctions between these constructs and the chosen variables. Autonomy refers to the sense of volition and self-endorsement when engaging in an activity. It involves acting in accordance with one’s own values and interests. Conversely, relatedness signifies the desire to connect and feel socially connected to others in the activity. While autonomy and relatedness are central psychological needs, their link to exercise intensity preference and tolerance is less direct than the connection with competence. Exercise intensity preference and tolerance pertain more closely to the practicalities and physical dimensions of the exercise experience. They involve aligning the actual intensity levels with an individual’s inclinations and physiological capabilities. In contrast, autonomy and relatedness are primarily concerned with the personal agency and social connection aspects of exercise participation. Exercise intensity preference and tolerance address the “how” of exercise, while autonomy and relatedness focus on the “why” and “with whom” aspects. Since our research objective was to explore how exercise intensity preference, tolerance, and competence jointly influence exercise intentions, it was not theoretically congruent to include autonomy and relatedness satisfaction and frustration in the same framework. The lack of alignment between these constructs and exercise intensity preference and tolerance would have introduced an unnecessary complexity to the study design, potentially obscuring the clarity of our investigation into the interplay between these specific variables. Therefore, by deliberately focusing on exercise intensity preference, tolerance, and competence, we aimed to offer a clear and well-defined examination of their impact on exercise intentions.

In the context of the Theory of Planned Behavior proposed by Ajzen [22], intentions refer to an individual’s conscious and deliberate decision to engage in a particular behavior, influenced by motivational and affective determinants [16,23]. In the realm of fitness, intentions encompass an individual’s premeditated resolve to partake in exercise activities based on their beliefs about the positive outcomes of such behavior, social influences, and their perceived ability to carry out the behavior. When individuals exhibit strong intentions to engage in fitness activities, they are more likely to translate these intentions into concrete actions, demonstrating higher levels of exercise commitment and participation [24].

### Current Study

Considering the practical implications of individuals’ exercise intensity preferences and tolerance, it becomes paramount to explore their influence on competence and subsequent behavioral intentions. Existing research highlights that aligning exercise intensity with personal preferences and tolerances positively impacts an individual’s sense of competence, ultimately influencing their intentions to persist in their fitness routines. Conversely, a lack of agreement between these factors might undermine individuals’ feelings of competence, potentially leading to decreased exercise intentions and reduced intentions. Furthermore, previous investigations frequently collapsed competence, autonomy, and relatedness into a holistic basic psychological needs factor [21], potentially diluting the unique impact of competence. This could be attributed to the theoretical alignment of competence with exercise intensity preference and tolerance, as explained earlier. Our study deliberately focuses solely on competence due to its foundational significance in the context of exercise intensity preference and tolerance, thereby enabling a targeted exploration of their nuanced associations. This decision is underscored by theoretical considerations and empirical evidence that highlight competence as the key mediator between preference, tolerance, and exercise intentions, while the roles of autonomy and relatedness in this context remain less pronounced [21,25].

The findings of this study could hold several important practical implications for fitness professionals, researchers, and practitioners. By investigating these relationships, we aim to provide empirical insights into how individual exercise preferences and physiological capacities intersect. This study contributes to the growing body of research that delves into the intricate dynamics of exercise intensity preference and tolerance [25]. Our focus on understanding how these factors shape exercise behavior is an important step towards a more comprehensive understanding of exercise adherence determinants. Additionally, the decision to specifically investigate individuals whose training regimens are aligned with their exercise intensity preference and tolerance is strategically grounded. By focusing on this subgroup, who are expected to have inherently higher exercise intentions due to their matched preferences, the study aims to pinpoint variables with stronger predictive power, thereby allowing for more effective strategies aimed at promoting adherence and long-term engagement in fitness activities.

The primary objective of this study is to comprehensively analyze the relationships between exercise intensity preference, tolerance, competence satisfaction, competence frustration, and exercise intentions within the fitness context. It is hypothesized that: (a) exercise intensity preference will be positively and significantly related to exercise intentions; (b) a positive and significant association between exercise intensity tolerance and exercise intentions will emerge; (c) competence satisfaction will positively and significantly relate to exercise intentions; (d) competence frustration will have a negative and significant relationship with exercise intentions; and (e) both competence satisfaction and competence frustration will mediate the relationships between exercise intensity preference, tolerance, and exercise intentions.

## 2. Materials and Methods

### 2.1. Participants

Prior to initiating the study, sample size calculations were executed using the Soper calculator for structural equation modeling [26]. The objective was to ascertain the optimal sample size essential for effectively testing the anticipated factor structure of the measurement model. Parameters such as the anticipated effect size (0.4), desired statistical power level (0.95), significance level (0.05), number of latent variables (4), and observed variables (1) were considered. The derived calculations indicated that a minimum sample size of 99 participants was deemed necessary to detect the expected effect size.

A total of 114 participants (76 females and 38 males) were included in this study. The participants’ ages ranged from 18 to 59 years (M = 33.23; SD = 10.542). On average, participants had a height of 166.02 cm (SD = 15.856) and weighed 68.02 kg (SD = 13.658). When asked about their gym experience, responses spanned from 5 to 312 months (M = 61.45; SD = 71.775).

The inclusion criteria for this study comprised individuals aged 18 years or older but below 65 years, who reported having their training regimen aligned with both their exercise intensity preference and tolerance levels (as indicated by checking the corresponding preference and tolerance boxes within the questionnaire; please refer to the data collection procedures below). Voluntary participation and an active gym attendance, involving at least one training session per week during the last month, were prerequisites for inclusion. These specified criteria were outlined at the outset of the questionnaire and within the consent form, ensuring the alignment of participants’ exercise experiences with the study’s objectives.

### 2.2. Data Collection Procedures

This study was conducted following the ethical approval of the institutional review board. After obtaining ethical clearance, outreach was made to multiple gyms and fitness centers to participate in the study, employing a convenience sampling strategy. Thorough explanations of the study’s aims and data collection methodologies were shared with club managers who granted their consent. Following this clearance, prospective participants were extended invitations via an internal email list, encouraging their voluntary involvement. Prior to participating, comprehensive information regarding the study’s objectives was provided to all participants, who then expressed their consent by checking a box before accessing a self-administered online questionnaire via a Google Forms link. The data collection process, which involved filling out the questionnaire, typically took less than 20 min on average.

### 2.3. Instruments

The present study utilized the Portuguese version of the Preference for and Tolerance of the Intensity of Exercise Questionnaire [27] which was comprised of ten items representing two scales corresponding to intensity preference (e.g., “The faster and harder the workout, the more pleasant I feel”) and intensity tolerance (e.g., “ Feeling tired during exercise is my signal to slow down or stop”) traits. Half of the items on each scale were scored inversely. Before data analysis, all negatively framed items were reversed to match the orientation of positively framed items. This process was carried out using IBM SPSS Statistics version 27 (IBM Corp., Armonk, NY, USA). Respondents were prompted to rate their beliefs and emotions during exercise on a 5-point bipolar Likert scale, ranging from 1 (“Strongly Disagree”) to 5 (“Strongly Agree”). This tool has demonstrated reliability and validity within the context of Portuguese fitness [28].

The present study utilized the Basic Psychological Needs Satisfaction and Frustration Scale Portuguese version [29]. This questionnaire consisted of 24 items organized into six dimensions, with four items allocated to each dimension. The dimensions encompass autonomy satisfaction, competence satisfaction, relatedness satisfaction, autonomy frustration, competence frustration, and relatedness frustration. For our specific investigation, we focused solely on the competence-related items, which were deemed relevant within the context of exercise and fitness. Composite factors were computed based on these selected items to represent overall competence satisfaction and frustration. Participants responded to all items using a 5-point scale ranging from 1 (“totally disagree”) to 5 (“totally agree”). This instrument has shown its reliability and validity in the realm of Portuguese fitness [30].

Intention was evaluated employing a validated Portuguese scale [31], grounded in the theory of planned behavior, to quantify intention toward future exercise. Three items gauge the intention to sustain exercise (e.g., “In the next 6 months, I will continue exercising as I do currently”) using a 7-point scale ranging from 1 (“Definitely not”) to 7 (“Definitely yes”). This tool has demonstrated reliability and validity within the context of exercise [32].

### 2.4. Statistical Analysis

Descriptive statistics were computed for all study variables using IBM SPSS Statistics version 27. Additionally, bivariate correlations were analyzed to explore the relationships between the variables in the study. The correlations provided insights into the strength and direction of the associations among the variables, enabling a preliminary understanding of the potential patterns and interdependencies present within the dataset. Using path analysis, a structural equation modeling analysis was performed in AMOS version 27 (IBM SPSS, Chicago, IL, USA), adhering to the methodological guidance provided by Hair et al. [33]. Given that the model was pre-specified, no model fit assessment was conducted, as path analysis specifies a fixed model a priori. The primary purpose of the structural model was to evaluate the relationships between the variables of interest, as supported by various theoretical frameworks. The Comparative Fit Index, Tucker–Lewis Index, Standardized Root Mean Square Residual, and Root Mean Square Error of Approximation were not assessed in this context, as the model was fixed based on established hypotheses and theoretical foundations [33,34]. The examination of direct and indirect regression coefficients aimed to scrutinize the study’s hypotheses. This entailed analyzing these coefficients to ascertain the direct relationships between the independent and dependent variables, while also evaluating the mediating impacts of the proposed intermediary variables [35].

## 3. Results

Table 1 presents the descriptive statistics. Both intensity traits exhibited scores above the midpoint of the scale, indicating a preference for higher exercise intensity and a corresponding tolerance. Competence satisfaction scores were above the midpoint, reflecting a positive perception of competence, while competence frustration scores were below the midpoint, indicating a lower degree of frustration. Additionally, intention scores were notably high. The key findings that support the study’s hypotheses are as follows: (a) a positive and significant correlation exists between exercise intensity preference and exercise intentions (r = 0.194, *p* = 0.039); (b) although not significant, there is a positive correlation between exercise intensity tolerance and exercise intentions (r = 0.088, *p* = 0.351); (c) a positive and significant correlation was observed between competence satisfaction and exercise intentions (r = 0.270, *p* = 0.004); (d) lastly, a negative and significant correlation was found between competence frustration and exercise intentions (r = −0.388, *p* < 0.001). For more details, see Table 1.

**Figure 1:**
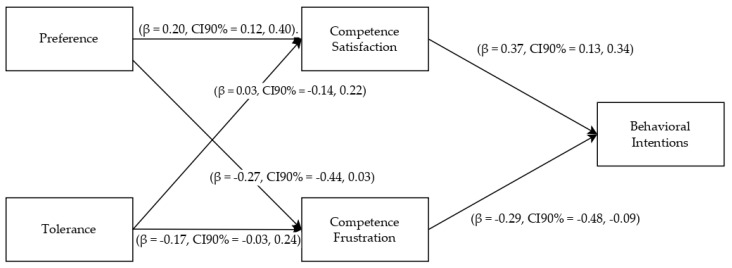
Path model (Notes: β = standardized beta coefficients; CI90% = Confidence Interval at 90%).

In the path analysis model, meaningful and positive correlations were identified among the constructs, with one notable exception: the relationship between exercise intensity tolerance and competence satisfaction, as highlighted in Figure 1. The findings further brought to light a series of direct effects operating among these constructs. Notably, exercise intensity preference demonstrated a positive link with competence satisfaction (β = 0.20, CI90% = 0.12, 0.40). Both intensity-related traits exhibited a negative but not significant connection with competence frustration (preference: β = −0.27, CI90% = −0.44, 0.03; tolerance: (β = −0.17, CI90% = −0.03, 0.24). It was observed that both competence satisfaction (β = 0.37, CI90% = 0.13, 0.34) and competence frustration (β = −0.29, CI90% = −0.48, −0.09) were significantly associated with individuals’ intentions. An indirect effect was noted between exercise intensity preference and exercise intentions (β = 0.11, CI90% = 0.03, 0.21). However, no such indirect effect was observed between exercise intensity tolerance and exercise intentions (β = 0.05, CI90% = −0.01, 0.14). In terms of the variance explained, it was determined that intentions to sustain the exercise regimen contributed to explaining 16% of the total variance.

## 4. Discussion

The objective of the present study was to comprehensively analyze the relationships between exercise intensity preference, tolerance, competence satisfaction, competence frustration, and exercise intentions within the fitness context. Based on the reported findings, the study largely supports the hypotheses, as exercise intensity preference positively relates to competence satisfaction. Both competence satisfaction and competence frustration significantly associate with exercise intentions. An indirect effect was noted between exercise intensity preference and exercise intentions. However, the hypothesized positive relationship between exercise intensity tolerance and exercise intentions, via mediators, was not supported.

Hypothesis A proposed a positive and significant relationship between exercise intensity preference and exercise intentions. Theoretically, this prediction aligns with the idea that individuals who have a greater preference for certain exercise intensities are more likely to enjoy their workouts and feel a sense of accomplishment, which could enhance their motivation to continue exercising [21]. Empirically, the findings of the study confirmed this hypothesis, as the analysis revealed a notable positive link between exercise intensity preference and competence satisfaction (β = 0.20, CI90% = 0.12, 0.40). This indicates that participants who exhibited a stronger preference for specific exercise intensities reported higher levels of satisfaction with their competence in performing those exercises. This empirical result provides support for the notion that a positive relationship exists between exercise intensity preference and a sense of competence.

Hypothesis B posited that there would be a positive and significant association between exercise intensity tolerance and exercise intentions. Theoretical reasoning for this hypothesis could be based on the idea that individuals with higher exercise intensity tolerance might be more likely to engage in challenging workouts, leading to greater improvements in fitness and subsequently higher intentions to continue exercising [21]. However, the empirical analysis yielded a non-significant association between exercise intensity tolerance and exercise intentions (β = 0.03, CI90% = −0.14, 0.22), which implies that the relationship between these variables was not statistically supported. Regarding the absence of a significant association between exercise intensity tolerance and competence satisfaction, several factors could contribute. It is possible that while exercise tolerance allows individuals to endure higher intensity workouts, satisfaction with competence might be influenced by factors beyond the mere ability to tolerate intensity. Competence satisfaction could depend on perceived skill mastery, accomplishment, and self-efficacy, which might not be solely determined by tolerance levels [27]. Additionally, individual differences in how tolerance and competence satisfaction are perceived and reported could also play a role in the lack of significant association observed in the study [28].

Hypothesis C proposed that there would be a positive and significant relationship between competence satisfaction and exercise intentions. Theoretically, this expectation is grounded in Self-Determination Theory, which postulates that feelings of competence and mastery in an activity positively influence intrinsic motivation and behavioral intentions [17,25]. It was anticipated that individuals who experience satisfaction in their exercise competence would demonstrate a greater inclination to continue their exercise regimen. The empirical analysis revealed results that align with the hypothesis (β = 0.37, CI90% = 0.13, 0.34). This finding indicates that competence satisfaction does indeed exhibit a significant positive association with exercise intentions. This empirical support substantiates the theoretical understanding that individuals who feel capable and accomplished in their exercise pursuits are more likely to harbor intentions to maintain their engagement [18,20].

Hypothesis D posited that a negative and significant relationship would exist between competence frustration and exercise intentions. The theoretical foundation of this hypothesis rests on the notion that feelings of frustration and inadequacy can undermine motivation and subsequently dampen exercise intentions [25]. It was expected that individuals encountering competence frustration would exhibit lower intentions to sustain their exercise behavior. The empirical results correspond with the theoretical expectations (β = −0.29, CI90% = −0.48, −0.09). The data indicate a significant negative relationship between competence frustration and exercise intentions, highlighting that individuals experiencing frustration in their exercise competence are indeed less likely to express intentions to continue their exercise regimen [32]. This underscores the detrimental impact of competence frustration on exercise intentions, reinforcing the need to address such negative emotions to promote adherence to exercise.

Hypothesis E aimed to investigate whether both competence satisfaction and competence frustration would serve as mediators in the relationships between exercise intensity preference, tolerance, and exercise intentions. The underlying theoretical framework suggests that an individual’s affective responses to their perceived competence can significantly influence their motivation and subsequent intentions to engage in a behavior, such as exercise [28]. The empirical results revealed interesting insights regarding these mediation effects. The study found an indirect effect between exercise intensity preference and exercise intentions, with need for competence acting as a significant mediator (β = 0.11, CI90% = 0.03, 0.21). This implies that individuals who possess a preference for a particular exercise intensity level are more likely to experience competence satisfaction, which, in turn, enhances their exercise intentions [16]. However, in the case of exercise intensity tolerance, the empirical analysis did not reveal a significant indirect effect on exercise intentions through the need for competence (β = 0.05, CI90% = −0.01, 0.14). This suggests that while exercise intensity tolerance might be an important personal trait for enduring challenging workouts, it does not exert its influence on exercise intentions through these specific psychological pathways [21]. These findings contribute to a nuanced understanding of the factors that mediate the relationships between exercise intensity preference, tolerance, and exercise intentions. While competence satisfaction appears to play a mediating role in the relationship between intensity preference and exercise intentions, the same cannot be said for intensity tolerance [28]. This underscores the complexity of the psychological mechanisms underlying exercise motivations and intentions, suggesting that different aspects of perceived competence may interact with different exercise-related traits to influence behavioral intentions.

### Limitations and Agenda for Future Research

The present study has several limitations that should be taken into consideration when interpreting the findings. Firstly, the use of cross-sectional data restricts our ability to establish causal relationships among exercise intensity preference, tolerance, competence satisfaction, competence frustration, and exercise intentions. To address this limitation, future research could employ longitudinal designs, enabling a more nuanced exploration of the causal directions between these variables over time. Secondly, our participant selection strategy focused solely on individuals whose training regimes aligned with their exercise intensity preference and tolerance. This approach might omit valuable insights from those who engage in exercises outside their preferred and tolerated intensity zones. Subsequent investigations could delve into the motivational dynamics and outcomes associated with such discrepancies. The study’s statistically significant findings were based on a relatively small sample size, potentially limiting the generalizability of the results and our ability to detect smaller effect sizes. Replicating the study with a larger and more diverse sample could enhance the robustness of the conclusions and facilitate exploration of potential subgroup variations. Furthermore, our sample predominantly consisted of Portuguese fitness gym-goers, which could restrict the applicability of the findings to broader populations with differing cultural and contextual backgrounds. Extending the study’s scope to encompass participants from diverse cultural contexts and various fitness settings would enable an exploration of potential cultural and contextual influences on the observed relationships. The inclusion of participants spanning a wide age range (18 to 59 years) introduces the possibility of age-related confounding variables that were not accounted for in the analyses. Future research could investigate potential age-related differences in the relationships under study, considering age as a moderating factor. Lastly, our study focused on experienced gym-goers, potentially overlooking the motivations and intentions of those new to exercise or with varying levels of experience. Expanding the investigation to include populations with differing exercise experience levels, including beginners and casual participants, would yield a more comprehensive understanding of the factors shaping exercise intentions.

## 5. Conclusions

Our findings are consistent with the theoretical framework that postulates the substantial influence of exercise intensity preference on shaping individuals’ intentions to partake in physical activity. The discerned positive correlation between exercise intensity preference and competence satisfaction underscores the significance of harmonizing workout intensity with individual inclinations, thereby fostering a sense of achievement and contentment. This outcome underscores the value of customizing exercise regimens according to personal preferences, potentially augmenting adherence to fitness routines. The theoretical implications of our investigation extend to the Self-Determination Theory, providing empirical substantiation for the pivotal role of competence satisfaction and its function in mediating the association between exercise intensity preference and exercise intentions. This finding reinforces the notion that individuals are more prone to intrinsic motivation when their psychological requisites for competence, autonomy, and connectedness are fulfilled. From a practical standpoint, our results hold notable consequences for fitness professionals, healthcare practitioners, and designers of exercise interventions. Tailoring exercise protocols to align with individuals’ intensity preferences can cultivate a sense of accomplishment and, by extension, amplify their intentions to persist in physical activity. Moreover, the recognition of the intermediary roles of competence satisfaction and frustration underscores the necessity of cultivating environments conducive to skill mastery and self-efficacy, while simultaneously mitigating factors contributing to frustration.

In conclusion, this study contributes significant insights to the domain of exercise psychology by elucidating the intricate network of interrelationships that influence exercise intentions. By highlighting the roles of exercise intensity preference, competence satisfaction, and competence frustration, we lay the groundwork for more efficacious interventions and strategies aimed at fostering enduring engagement in physical activity. As we progress, addressing of the identified limitations and the advancement of these findings will assuredly enhance our comprehension of the motivational dynamics inherent in the pursuit of a healthier and more active lifestyle.

## Figures and Tables

**Table 1:** Descriptive statistics and correlation analysis.

Variables	M	SD	S	K	1	2	3	4
1. Preference	17.20	4.17	−0.54	−0.06	1			
2. Tolerance	16.04	3.81	0.05	−0.53	0.52 **	1		
3. Competence Satisfaction	3.67	0.62	−0.91	2.05	0.27 **	0.20 *	1	
4. Competence Frustration	1.97	0.80	0.79	0.47	−0.36 **	−0.31 **	−0.71 **	1
5. Intentions	5.03	1.21	−0.94	−0.38	0.19 *	0.09	0.27 **	0.19 *

Notes: M = Mean; SD = Standard Deviation; S = Skewness; K = Kurtosis; ** *p* < 0.01; * at *p* < 0.05.

## Data Availability

The data used in this study were obtained under a specific license exclusively for the purposes of this research. While the data supporting the findings of this study are not publicly available, they can be requested and accessed upon reasonable inquiry, subject to permission from the Life Quality Research Center and the corresponding author.
